# Evaluating the role of bacterial diversity in supporting soil ecosystem functions under anthropogenic stress

**DOI:** 10.1038/s43705-023-00273-1

**Published:** 2023-07-03

**Authors:** Ernest D. Osburn, Gaowen Yang, Matthias C. Rillig, Michael S. Strickland

**Affiliations:** 1 Department of Soil and Water Systems, University of Idaho, Moscow, ID USA; 2 College of Grassland Science and Technology, China Agricultural University, 100193 Beijing, China; 3 Institute of Biology, Freie Universität Berlin, 14195 Berlin, Germany; 4 Berlin-Brandenburg Institute of Advanced Biodiversity Research (BBIB), 14195 Berlin, Germany

**Keywords:** Microbial ecology, Molecular ecology, Biogeochemistry, Community ecology

## Abstract

Ecosystem functions and services are under threat from anthropogenic global change at a planetary scale. Microorganisms are the dominant drivers of nearly all ecosystem functions and therefore ecosystem-scale responses are dependent on responses of resident microbial communities. However, the specific characteristics of microbial communities that contribute to ecosystem stability under anthropogenic stress are unknown. We evaluated bacterial drivers of ecosystem stability by generating wide experimental gradients of bacterial diversity in soils, applying stress to the soils, and measuring responses of several microbial-mediated ecosystem processes, including C and N cycling rates and soil enzyme activities. Some processes (e.g., C mineralization) exhibited positive correlations with bacterial diversity and losses of diversity resulted in reduced stability of nearly all processes. However, comprehensive evaluation of all potential bacterial drivers of the processes revealed that bacterial α diversity *per se* was never among the most important predictors of ecosystem functions. Instead, key predictors included total microbial biomass, 16S gene abundance, bacterial ASV membership, and abundances of specific prokaryotic taxa and functional groups (e.g., nitrifying taxa). These results suggest that bacterial α diversity may be a useful indicator of soil ecosystem function and stability, but that other characteristics of bacterial communities are stronger statistical predictors of ecosystem function and better reflect the biological mechanisms by which microbial communities influence ecosystems. Overall, our results provide insight into the role of microorganisms in supporting ecosystem function and stability by identifying specific characteristics of bacterial communities that are critical for understanding and predicting ecosystem responses to global change.

## Introduction

Soils are the foundation of terrestrial ecosystems and support many ecosystem functions and services, including plant productivity, carbon storage, and nutrient cycling [1]. These functions are performed primarily by microorganisms (e.g., bacteria, fungi) that inhabit soil environments [2]. However, soil ecosystems and the microbial communities they host are experiencing intensifying stress associated with anthropogenic activities, including land use change, climate change, and agricultural inputs (e.g., fertilizers, pesticides, antibiotics) [3, 4]. These anthropogenic stressors are destabilizing terrestrial ecosystems globally [3, 5]. Given the key role of microorganisms in underpinning ecosystem functions, it is imperative to understand how microbial communities influence ecosystem stability under anthropogenic stress.

Ecosystem stability can be defined as the ability of an ecosystem to defy change or return to equilibrium following a disturbance [6]. The stability of ecosystems is thought to be dependent upon the diversity and composition of the resident biotic communities [6, 7]. This relationship is well studied in plant communities, where species diversity has been shown to buffer ecosystem productivity against anthropogenic global change drivers, including heat stress, drought, and nutrient additions [8, 9]. In contrast, the role of microorganisms in contributing to ecosystem stability is poorly understood. This is due in part to the exceptional diversity of microbial communities [10] along with the fact that microorganisms are difficult to observe and have been historically neglected in biodiversity surveys [11]. As a result, relationships among anthropogenic stress, microbial communities, and ecosystem stability are uncertain.

Indeed, rigorous evaluations of relationships between microbial communities and the ecosystem-scale processes they facilitate have only started to emerge in the past decade – these studies have shown soil microbial α diversity to be positively associated with several ecosystem functions, including C mineralization [12], C use efficiency [13], denitrification [14], plant productivity [15], and overall ecosystem functioning, i.e., multifunctionality [16–18]. In contrast, other studies have shown that composition of the constituent microbial taxa (i.e., community ‘membership’) to be more important than diversity *per se* in accounting for variation in ecosystem functions [4, 19, 20]. Regardless, it is now clear that ecosystem functions can be attributed to specific, measurable characteristics of the microbial communities that inhabit those environments. Because of the importance of microorganisms in facilitating ecosystem functions, microbial communities will also be important in stabilizing ecosystems against anthropogenic stress. However, specific characteristics of microbial communities that confer ecosystem stability are unknown. Indeed, very few studies have explicitly examined stability of ecosystem processes (e.g., respiration) across microbial community gradients [21–23], and those that do exist focused on influences of microbial α diversity and not other microbial community characteristics that may influence ecosystem stability, e.g., microbial biomass, functional group abundances, community membership.

Though it is difficult to generate specific predictions regarding microbial contributions to ecosystem stability, we note that microbial diversity and community membership are highly variable among ecosystem types [24, 25], and therefore different ecosystems are likely to exhibit distinct microbial-mediated ecosystem stress responses. This high natural variation in microbial communities is compounded by the fact that communities are already being influenced by anthropogenic stress globally. For example, warming, nitrogen (N) fertilization, and increased salinity all have reduced soil microbial α diversity in multiple ecosystems [26–28], and, similar to plants, these lower diversity communities may result in reduced ecosystem stability following additional stress. In this study, we experimentally generate wide gradients in soil bacterial diversity, induce stress in those soils, and then measure many soil ecosystem processes, with the goal of identifying specific characteristics of bacterial communities that confer stability.

Our study includes soils from contrasting ecosystems (undisturbed prairie vs. cultivated field) that host dramatically different soil bacterial communities (Supplementary Fig. S1). We generated additional variation in bacterial communities using sterilization-dilution and induced stress in the soils using oxytetracycline, a widely used agricultural antibiotic [29, 30]. We applied this particular stressor because antibiotics are a contaminant that enter both agricultural and natural soils via multiple routes, e.g., direct application of manure, runoff, wind deposition [29, 31]. We then measured many microbial-mediated soil ecosystem processes, including rates of C and N cycling, soil enzyme activities, and overall biogeochemical functioning (i.e., multifunctionality). Finally, we quantified the importance of bacterial community characteristics in accounting for variation in ecosystem functions using random forest modeling. To enhance the external validity to our findings, we additionally applied our analytical approach to data from a previous study from another continent that used a similar dilution approach and also exposed soils to anthropogenic stressors (up to ten, including antibiotics) [23]. We hypothesized that, like plant communities, soils with higher bacterial diversity would exhibit greater stability of ecosystem functions following application of stress.

## Material and methods

### Soil sampling and experimental design

We collected soils from two contrasting ecosystems that vary in management history, soil properties, and resident microbial communities (Supplementary Fig. S1, Supplementary Table S1). The first sampling site was the Dave Skinner Preserve in Moscow, ID (46°40′ 39.8“N, 116°58′ 36.1“W), an undisturbed prairie remnant characterized by a diverse native plant community comprised primarily of herbaceous plants, small shrubs, and bunchgrasses (e.g., *Pseudoroegneria spicata*). The prairie site is at an elevation of 1128 m and receives 685.8 mm of precipitation per year. The second site was a cultivated field at the NRCS Plant Materials Science Center in Pullman, WA (46° 43’ 21.3276“N, 117° 8’ 27.9198“W), which is at an elevation of 767 m and receives 516.9 mm of precipitation per year. This site has a legacy of N fertilization and is intensively managed via annual tillage and a winter wheat-fallow crop rotation. The cultivated soils have lower pH, higher NO_3_-N, lower NH_4_-N, and lower C:N compared with the undisturbed prairie soils (Supplementary Table S1). In both sites, soil was collected as ten 0–10 cm depth cores and composited by site. The composite samples were sieved (4 mm) and stored at 4 °C.

A 1 kg subsample of both soils was sterilized by 40 kGy gamma radiation. Sterility was verified by monitoring microbial activity (i.e., respiration) of the irradiated soils. Sterilized soils (30 g dry weight) were added to autoclaved mason jars and sterile microcosms were inoculated with suspensions originating from the same nonsterile soil. Inoculum suspensions were extracted by shaking 6 g of nonsterile soil in 50 ml sterile 1X PBS solution for 1 h, with the two soils extracted in quadruplicate and then the replicates pooled. To create a gradient of diversity, three levels of dilution were then used as inocula: undiluted suspension (D0), 1 × 10^−3^ diluted suspension (D1), and 1 × 10^−6^ diluted suspension (D2). We adjusted soils to 65% of water holding capacity and incubated soils at 20 °C for six weeks to allow for microbial establishment. At the end of the six weeks, we verified that microbial activity had stabilized in all microcosms by measuring respiration using a LI-COR 8100 A (LI-COR Biosciences, Lincoln, Nebraska, USA). The respiration data showed that the dilution treatments within each land use had indistinguishable microbial activity (Supplementary Fig. S2), indicating that biomass recovery had likely occurred at this time.

After community establishment, we induced stress in communities by adding oxytetracycline, an agricultural antibiotic used in livestock production and commonly found in soils [29, 30]. We added oxytetracycline once weekly for one month at a rate of 50 µg g soil^−1^, which is within the range detected in soils [29]. Therefore, our treatments represent realistic levels of stress experienced by soil communities. Control soils received an equal volume of sterile water. Each land use × microbial diversity × stress treatment was replicated 5 times (2 land uses × 3 diversity levels × 2 stress levels × 5 replicates = 60 experimental units). As additional controls, we also incubated 5 replicates of both original nonsterile soils along with 5 replicates of both sterilized soils that were inoculated with only sterile water, resulting in a grand total of 80 experimental units.

### Microbial community analyses

At the conclusion of the experiment, we measured several bacterial community characteristics in soils. We measured total microbial biomass in all soils using a chloroform extraction technique [32] and as an additional metric of microbial biomass, we extracted DNA from samples using the Qiagen PowerSoil kit (Qiagen, Valencia, CA, USA) and quantified DNA yield using a Qubit fluorometer (Thermo Fisher Inc., Waltham, MA, United States) [12, 21]. We quantified total bacterial and fungal abundance in samples by qPCR amplification of the 16S rRNA gene and ITS region, respectively [33]. The ITS qPCR data indicated extremely low fungal abundance in the sterilized-inoculated soils, particularly the D1 and D2 treatments (Supplementary Fig. S3). Because of the apparent lack of fungal establishment in these microcosms, likely because of absence (or very low abundance) of fungal cells in the inocula, we chose to not consider fungal communities any further. We also used qPCR to quantify the abundance of nitrifying microorganisms, i.e., ammonia-oxidizing bacteria (AOB) and archaea (AOA) [34, 35], as well as two tetracycline antibiotic resistance genes (ARGs): tetW and tetM [36, 37]. Complete information on qPCR assays is provided in the Supplementary information.

We characterized bacterial communities by amplicon sequencing of the V4 region of the 16S rRNA gene using the 515 F/806 R primer pair [38]. Amplicons were sequenced on an Illumina MiSeq using 250 bp paired-end reads. Raw reads were deposited in the NCBI archive under accession number PRJNA853373. Raw sequences were processed with DADA2 [39] and taxonomy was assigned to the processed sequences (i.e., amplicon sequence variants, ASVs) using a soil-specific naïve Bayes classifier [40] trained on the SILVA database (version 138.1) [41]. We calculated bacterial α diversity metrics (Shannon index, ASV richness) following repeated rarefaction (1000 iterations), a robust method of accounting for variation in sequence depth among samples [42]. We repeatedly rarefied at a sequence depth of 15,418, which rarefaction curves indicated to be adequate coverage for our samples (Supplementary Fig. S4). We identified bacterial genera that were differentially abundant among the diversity and stress treatments using DESeq2 [43]. As an indicator of community-level bacterial life history traits (e.g., growth rates, nutrient use), we estimated average 16S rRNA operon number for each community using rrnDB [44]. Detailed information on all sequencing methods is provided in the Supplementary Information.

### Microbial-mediated ecosystem processes

At the end of the experiment, we measured basal respiration rates (i.e., C mineralization) of all soils using a 24-hour static incubation method [45] and we simulated soil responses to labile C inputs (e.g., root exudates) using substrate-induced respiration (SIR) [46]. As an index of microbial efficiency, we calculated a metabolic quotient for each sample (i.e., biomass-specific respiration, qCO_2_) [47]. To measure net N mineralization rates, we calculated the rate of total inorganic-N (NH_4_-N + NO_3_-N) accumulation in the soils [48–50] and to measure net nitrification rates we calculated the rate of NO_3_-N accumulation [48]. We also measured rates of four hydrolytic extracellular enzymes involved in C cycling (β-glucosidase), N cycling (leucine aminopeptidase and N-acetyl-β-glucosaminidase), and phosphorus cycling (acid phosphatase) using a fluorometric microplate method [51]. Because the four enzymes exhibited generally similar patterns across treatments (Supplementary Fig. S5), as an index of overall enzymatic function, we summed the four rates to calculate total enzyme activity for each sample [52]. To quantify stability of the above processes, we used the ‘resistance’ index of Orwin and Wardle [53] where resistance = 1 − (2 | *D*_0_ | /(*C*_0_ + | *D*_0_ | )). *D*_0_ represents the value of stressed soils while *C*_0_ is the value of controls. This index is bounded between −1 and 1 with a value of ‘1’ indicating no change in an ecosystem process following stress. To assess overall biogeochemical ecosystem function, i.e., multifunctionality, we conducted multivariate analyses considering all the microbial-mediated functions (see below).

### Data analyses

**Fig. 1:**
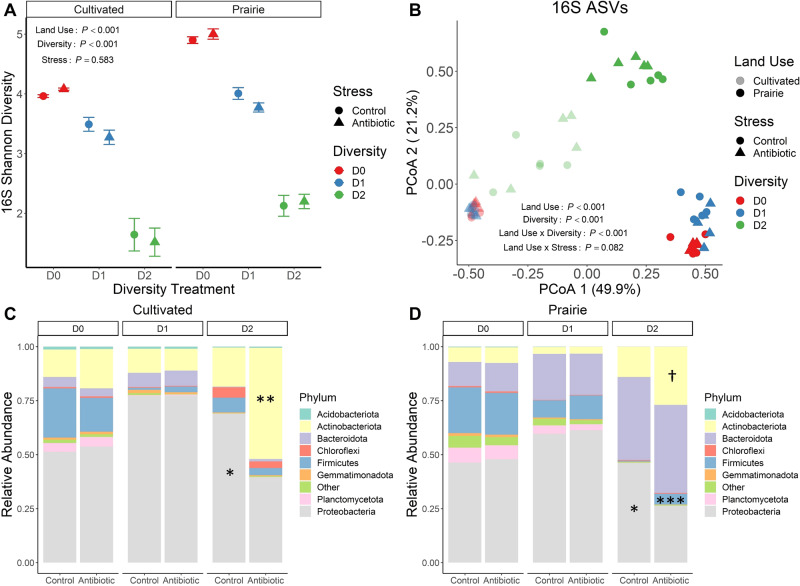
Effects of land use, diversity, and stress treatments on bacterial α diversity and community membership. Panels show bacterial α diversity (**A**), 16S ASV membership (**B**), and relative abundances of bacterial phyla in the cultivated (**C**) and prairie (**D**) ecosystems. The PCoA ordination in (**B**) is based on the RC_Bray_ dissimilarity matrix. In (**A**), symbols represent treatment means while error bars represent the standard error of the mean. *P* values in (**A**) are from (generalized) linear models and in (**B**) are from permutational analysis of variance (PERMANOVA) based on RC_Bray_ dissimilarity (**B**). Asterisks in (**C**) and (**D**) indicate significant pairwise differences in relative abundance (Tukey method) between stress treatments within a particular land use × diversity combination at the following levels: † *P* < 0.1, * *P* < 0.05, ** *P* < 0.01, *** *P* < 0.001. Diversity treatments are as follows: inoculated with undiluted soil suspension (D0), 1 × 10^−3^ diluted suspension (D1), and 1 × 10^−6^ diluted suspension (D2).

All statistical analyses were performed in R [54]. For all univariate analyses we determined effects of land use, diversity (i.e., dilution) treatment, and stress (i.e., antibiotic) using linear models (‘lm’ function) or generalized linear models (‘glm’ function, Gamma distribution, log-link) when linear models did not meet assumptions of normality of residuals. We tested for pairwise differences between control and stress treatments within each land use × diversity combination using the ‘contrast’ function (Tukey method) in the emmeans R package [55]. We performed multivariate analysis of bacterial communities and ecosystem multifunctionality using the vegan R package [56]. Because our experiment involved large changes in α diversity (Fig. 1A) and because typical dissimilarity metrics are confounded with α diversity [57], we used a Raup-Crick null model to generate a dissimilarity matrix that is independent of differences in α diversity between samples. The Raup-Crick approach standardizes the observed dissimilarity matrix (Bray-Curtis) against probabilistically assembled null communities (1000 iterations) where the overall relative abundance of each ASV and ASV richness for each sample are held constant [57, 58]. We used the resulting RC_Bray_ dissimilarity matrix for all downstream analyses. We visualized community composition using principal coordinates analysis (PCoA, ‘cmdscale’ function) and determined effects of land use, dilution, and stress using PERMANOVA (‘adonis2’ function). We visualized multifunctionality by performing principal components analysis on the scaled rates of the individual processes (‘princomp’ function) and determined effects of land use, diversity treatment, and stress on multifunctionality using PERMANOVA with Euclidean distances.

We quantified the importance of microbial variables in accounting for variation in ecosystem process rates using random forest regression (randomForest R package) [59]. The random forest model for each process rate was tuned such that the number of predictors randomly sampled as candidates at each split minimized the out-of-bag error rate of the model. All random forest models were run with 10,000 trees. The ‘Importance’ of predictor variables was calculated by determining the increase in model error after randomly shuffling each candidate predictor across the data set. For each ecosystem process, we considered a range of potential microbial predictors: 16S ASV richness, 16S Shannon diversity, ASV membership (i.e., PCoA axis scores), total microbial biomass, 16S gene abundance, average 16S operon numbers, and tetracycline ARG abundances. For nitrification rates, the abundances of nitrifying organisms (AOA, AOB) were also considered as candidate predictors. To explore influences of broad taxonomic shifts on ecosystem processes, we also considered Proteobacteria:Actinobacteria ratios, as these two dominant lineages exhibited opposite responses to stress in our study (Fig. 1C, D). While we recognize that functional traits of specific bacterial taxa cannot be predicted based on their phylum-level taxonomy [60], several prior studies have associated Proteobacteria and Actinobacteria with different community-scale functioning [19, 61, 62]. To determine if finer-scale taxonomic information improved model results, we ran alternative random forest models using the relative abundances of six genera identified by DESeq2 to be differentially abundant among our treatments. For the random forest model with multifunctionality as a response variable, we calculated multifunctionality for each sample by scaling each of the individual rates and calculating the mean scaled rate for each sample [16, 19].

For external validation, we applied the same analytical approach to data from a recent study that also examined ecosystem responses to anthropogenic stressors across microbial diversity gradients and included similar ecosystem functions (e.g., C mineralization, enzyme activities) and microbial variables (e.g., bacterial abundance, α diversity and membership) to our experiment but also included fungal abundance, α diversity, and membership metrics [23].

## Results

### Effects on microbial communities

Our sterilization-dilution scheme successfully reduced soil bacterial α diversity – on average, D1 (1 × 10^−3^ diluted) soils had 19% lower 16S Shannon diversity than D0 soils while D2 (1 × 10^−6^ diluted) soils had 49% lower Shannon diversity than D1 soils and 58% lower diversity than D0 soils (Fig. 1A). This corresponded to a loss of 178 bacterial taxa from D0 to D1 on average (46% loss in richness) and an additional 92 taxa from D1 to D2 on average (45% additional loss in richness, Supplementary Fig. S6). Shannon diversity was also 15% higher in prairie soils than cultivated soils on average (Fig. 1A). Stress application (i.e., antibiotic) did not affect bacterial α diversity at any level of dilution (Fig. 1A). The diversity treatments also significantly altered 16S ASV community membership independently of α diversity, though the effects differed between land uses – in the prairie soils, all diversity treatments were distinct, while in the cultivated soils D2 formed a distinct cluster from D0 and D1 (Fig. 1B). In general, lower diversity communities exhibited increasing dissimilarity from the original nonsterile soil communities but remained distinct from sterile negative controls (Supplementary Fig. S7). Antibiotic stress only marginally affected 16S ASV membership and only in the prairie soils (land use × stress interaction, Fig. 1B), likely because we used low, environmentally relevant concentrations of the antibiotic.

Though communities showed only minor responses to antibiotic stress at the ASV level, aggregating sequences at higher taxonomic levels revealed clear responses (Fig. 1C, D). Notably, these bacterial community responses to stress only occurred in low diversity soils (1 × 10^−6^ diluted, D2) (Fig. 1C, D). For example, stress reduced abundance of Proteobacteria in the D2 soils by 43% on average (Fig. 1C, D). Antibiotic stress also promoted Gram positive taxa in D2 soils – relative abundance of Actinobacteria was 142% higher in stressed D2 soils (Fig. 1C, D), while in the prairie ecosystem, relative abundance of Firmicutes was >9-fold higher in the stressed D2 soils (Fig. 1D). Further, DESeq2 identified six abundant genera to be responsive to our treatments: *Pseudomonas*, *Bacillus*, *Arthrobacter*, *Flavobacterium*, *Massilia*, and *Rhodanobacter*. Similar to the phylum-level results, these genera exhibited stress responses only in reduced diversity soils – for example, in D2 soils, antibiotics reduced the relative abundances of *Bacillus* and *Pseudomonas* by 71% and 97%, respectively, and increased the relative abundance of *Arthrobacter* by 109% (Supplementary Figs. S8, S9). Similar to the taxonomic responses, microbial biomass only responded to stress in D2 soils, though biomass unexpectedly increased by 69% in those soils (Supplementary Fig. S10). This could be due to bacterial production of defense compounds (e.g., exo-polymeric substances, EPS) in response to the antibiotic additions [63]. 16S gene copy abundance did not exhibit stress responses (Supplementary Fig. S10).

### Effects on microbial-mediated ecosystem processes

**Fig. 2:**
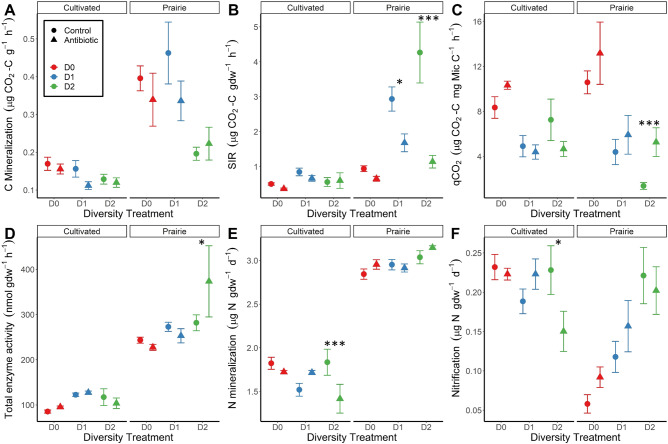
Effects of land use, diversity, and stress treatments on six microbial-mediated soil ecosystem functions. Panels show C mineralization (**A**) potential microbial activity (SIR) (**B**), metabolic quotient (qCO_2_) (**C**), total hydrolytic enzyme activity (**D**), N mineralization (**E**), and nitrification (**F**). Symbols represent treatment means while error bars represent the standard error of the mean. Asterisks indicate significant pairwise differences (Tukey method) between stress treatments within a particular land use × diversity combination at the following levels: *P < 0.05, **P < 0.01, ***P < 0.001. Diversity treatments are as follows: inoculated with undiluted soil suspension (D0), 1 × 10^−3^ diluted suspension (D1), and 1 × 10^−6^ diluted suspension (D2).

**Fig. 3:**
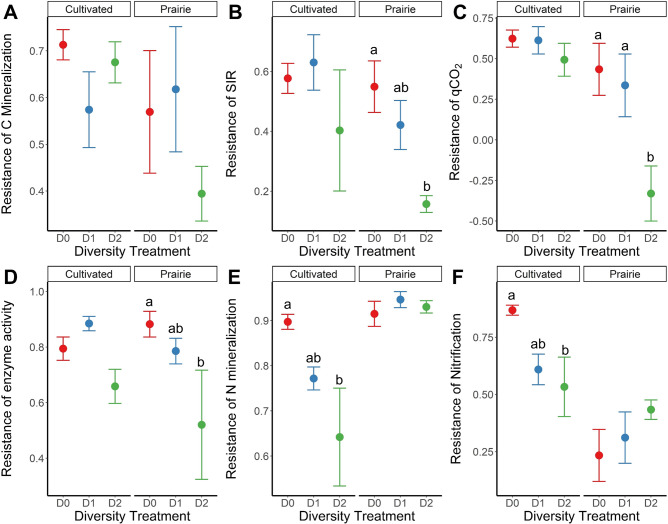
Stability of ecosystem functions calculated according to the ‘resistance’ index of Orwin and Wardle [53] where resistance = 1 − (2 | *D*_0_ | /(*C*_0_ + | *D*_0_ | )). *D*_0_ represents the value of stressed soils while *C*_0_ is the value of controls. Panels show the resistance of C mineralization (**A**), potential microbial activity (SIR) (**B**), metabolic quotient (qCO_2_) (**C**), total hydrolytic enzyme activity (**D**), N mineralization (**E**), and nitrification (**F**). Symbols represent treatment means while error bars represent the standard error of the mean. Lower values indicate reduced stability. Different letters indicate significant pairwise differences in resistance between diversity treatments within a particular land use (*P* < 0.05, Tukey method). Diversity treatments are as follows: inoculated with undiluted soil suspension (D0), 1 × 10^−3^ diluted suspension (D1), and 1 × 10^−6^ diluted suspension (D2).

Antibiotic stress influenced nearly all microbial-mediated ecosystem process rates (Fig. 2) – of all measured ecosystem functions, only C mineralization rates exhibited no stress responses (Fig. 2A). However, similar to effects on bacterial taxa, stress only affected ecosystem functions in reduced diversity soils, particularly the lowest diversity D2 soils (Fig. 2). For example, stress application reduced substrate-induced respiration (SIR) by 64% on average in D2 soils (Fig. 2B). In the prairie D2 soils, we also observed 3.8-fold higher biomass-specific respiration (qCO_2_) and 25% higher enzyme activity in stressed than control treatments (Fig. 2C, D). N-cycle processes were also affected by stress. N mineralization rates were 23% lower in the cultivated D2 soils under stress (Fig. 2E) and nitrification rates were 21% lower on average in stressed D2 soils (Fig. 2F). The nitrification responses reflect reduced abundance of nitrifying bacteria (AOB) and nitrifying archaea (AOA), which were 25% and 29% lower in abundance, respectively, in stressed D2 soils (Supplementary Fig. S11). In addition to analyzing effects of stress treatments on the ecosystem process rates directly, we quantified stability of each process using a ‘resistance’ index [53]. This analysis confirmed that all ecosystem functions other than C mineralization exhibited reduced stability (i.e., significantly lower resistance) in low diversity D2 soils (Fig. 3).

### Relationships between microbial communities and ecosystem processes

**Fig. 4:**
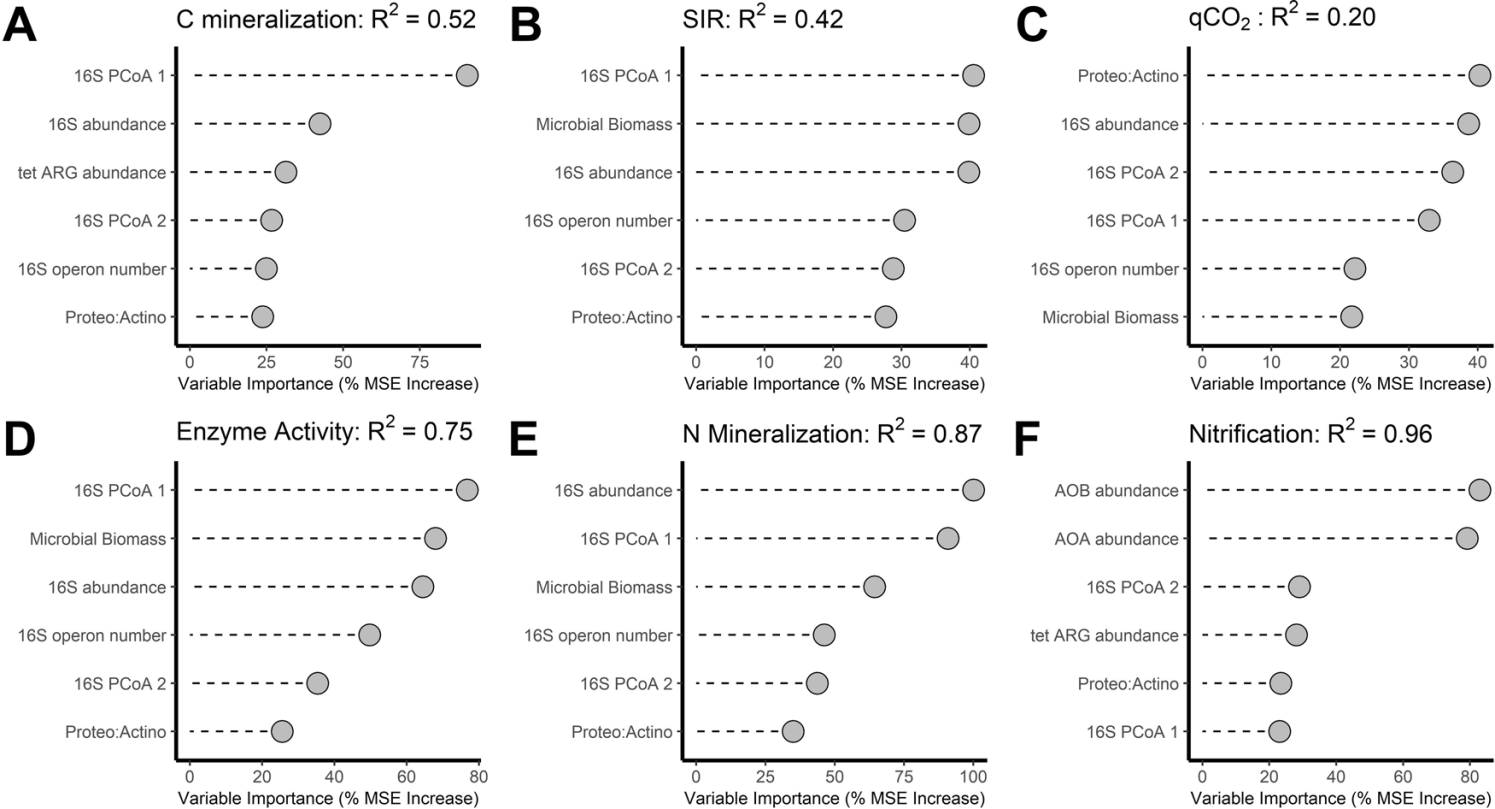
Random forest models for each soil ecosystem function. Shown are the top six microbial predictors for C mineralization (**A**) potential microbial activity (SIR) (**B**), metabolic quotient (qCO_2_) (**C**), hydrolytic enzyme activity (**D**), N mineralization (**E**), and nitrification (**F**). The ‘Importance’ of predictor variables was calculated by determining the increase in model error after randomly shuffling each candidate predictor across the data set. The random forest model for each process rate was tuned such that the number of predictor variables randomly sampled as candidates at each split minimized the out-of-bag error rate of the model. All random forests models were run with 10,000 trees. Models for SIR and qCO2 were improved by including genus-level taxonomic information, shown on Supplementary Fig. S14.

Bacterial α diversity metrics were significantly correlated with some ecosystem process rates – for example, 16S Shannon diversity was positively correlated with C mineralization while 16S ASV richness was positively correlated with C mineralization and N mineralization rates (Supplementary Figs. S12, S13). However, in contrast to expectations, random forest regression revealed that microbial α diversity metrics were never among the strongest statistical predictors of ecosystem function, despite the large variation in α diversity among our treatments and despite the presence of significant correlations between α diversity and functions (Fig. 4). Indeed, C mineralization, SIR, and enzyme activity were all best predicted by bacterial ASV membership, i.e., PCoA axis 1 (Fig. 4A, B, D), which was quantified independently of α diversity and along which communities varied according to land use and diversity treatments (Fig. 1B). Taxonomic shifts were the best predictor of qCO_2_, where higher qCO_2_ (i.e., lower efficiency) was associated with lower Proteobacteria:Actinobacteria ratios (Fig. 4C). For nitrification rates, abundance of nitrifying taxa was the most important predictor (Fig. 4F). For most processes, microbial abundance metrics (i.e., biomass, 16 S abundance) were also among the most important predictors, as were microbial life history indicators (i.e., average 16 S operon number) (Fig. 4). Alternative random forest models containing differentially abundant genera as predictors improved predictions for SIR and qCO_2_ – SIR was strongly positively linked to *Pseudomonas* while *Bacillus* relative abundance was a strong predictor of qCO_2_ (Supplementary Fig. S14).

### Multifunctionality

**Fig. 5:**
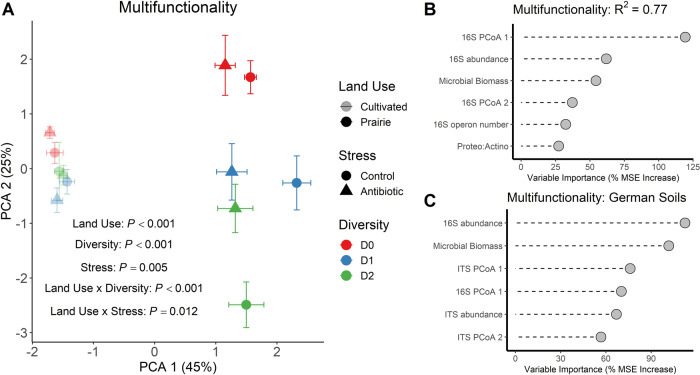
Ecosystem multifunctionality and its microbial drivers. Multifunctionality is represented by principal components analysis of all six soil functions (**A**). *P* values in (**A**) are from PERMANOVA with Euclidean distances. Symbols represent centroids while error bars represent one standard error of the mean. For the random forest regression model with multifunctionality as the response (**B**), multifunctionality was calculated by scaling each of the individual ecosystem functions and calculating the mean scaled rate for each sample. Diversity treatments are as follows: inoculated with undiluted soil suspension (D0), 1 × 10^−3^ diluted suspension (D1), and 1 × 10^−6^ diluted suspension (D2). (**C**) displays the random forest regression model with multifunctionality from Yang et al. 2022 as the response (R^2^ = 0.56). Models for each of the nine individual functions are provided on Supplementary Fig. S15. Models were run as described on Fig. 4 and multifunctionality was calculated as described in (**B**).

As a final assessment of ecosystem stress responses across our microbial community gradients, we determined responses of ecosystem multifunctionality, i.e., responses of all six biogeochemical processes considered simultaneously. This analysis showed that the cultivated and prairie soils were functionally distinct and that the prairie soils were overall more responsive to changes in diversity and stress treatments compared with the cultivated soils (Fig. 5A). Further, our random forest model with multifunctionality as a response confirms the results from individual functions – that bacterial community membership and abundance, and not α diversity, emerge as the strongest statistical predictors of soil ecosystem function (Fig. 5B).

### Re-analysis of a prior microbial diversity—ecosystem stress experiment

To seek additional support for these results, we applied our analytical approach to data from another recent study, using soils from northern Germany, that also examined effects of global change stressors on soil ecosystem functions across microbial diversity gradients [23]. This study measured similar ecosystem processes to ours (e.g., C mineralization, enzyme activities) but examined a range of different anthropogenic stressors (e.g., warming, drought, salinity) as well as number of stressors (up to 10 applied simultaneously). Re-analysis of this data set revealed similar results to ours – that ecosystem responses were best explained by microbial membership and abundance metrics, while microbial α diversity was not a strong statistical predictor (Fig. 5C). This pattern held when examining functions individually (Supplementary Fig. S15) and when combining all functions into a multifunctionality index (Fig. 5C). The German experiment also revealed the importance of fungal community membership for some functions, e.g., phosphatase enzyme activity (Supplementary Fig. S15).

## Discussion

Our results clearly demonstrate the importance of microbial abundance and bacterial community membership in driving ecosystem functioning and stability, whereas bacterial α diversity was not a strong predictor of the measured processes. Importantly, while some microbial metrics may be confounded with α diversity, the most consistently important microbial variable in our study was ASV-level community membership (i.e., PCoA scores), which we quantified independently of α diversity. This is striking because, at face value, our experimental results do suggest strong influences of α diversity; stress responses of ecosystem processes were only seen in low diversity treatments and we also observed significant bivariate relationships between bacterial α diversity and some processes, e.g., between Shannon diversity and C mineralization rates. Multiple prior studies have inferred mechanistic importance of microbial α diversity to ecosystem functioning on the basis of similar observations [12–14, 16**–**18, 21]. Our results demonstrate the limitations of such inferences – changes in microbial α diversity are likely to be accompanied by other changes to microbial communities, e.g., community membership, which may also involve shifts in microbial life history strategies or specific functional groups. We show that these microbial community membership and abundance characteristics, rather than α diversity, most strongly predict ecosystem functions and therefore are more likely to represent the direct microbial drivers of functional changes at the ecosystem scale. This conclusion is also supported by the fact that abundance metrics (e.g., 16S abundance, total biomass) were not correlated with bacterial α diversity metrics in our study (Supplementary Fig. S13), indicating that influences of abundance were not confounded with α diversity. These results support prior work that found stronger relationships between microbial community composition and ecosystem function compared with microbial α diversity [4, 19, 20].

Some prior studies focused on plant communities have similarly found ecosystem functioning to be incorrectly attributed to plant α diversity when other characteristics of plant communities (e.g., biomass, functional groups) were omitted from analyses [64, 65]. Indeed, a recent analysis demonstrated that plant diversity – ecosystem function relationships are noncausal associations and that ecosystem functions are instead driven by functional traits of the resident species [66]. We suggest that similar misattribution of α diversity effects may also occur in microbial studies if other community responses that accompany diversity changes are not considered. This consideration is particularly important in extraordinarily diverse soil microbial communities – indeed, even our dramatically reduced diversity D2 communities hosted ~100 unique bacterial ASVs, an α diversity level much greater than that seen in studies focused on macrobiological biodiversity [8, 9, 67].

Though α diversity was not a strong predictor of ecosystem processes, we did observe that specific bacterial taxa only exhibited stress responses in low diversity treatments, which suggests that diversity begets compositional stability of communities under stress. This result suggests indirect influences of bacterial α diversity on ecosystem function and stability. For example, it is likely that α diversity indirectly supported soil function and stability in our experiment via increased functional redundancy in the more diverse bacterial communities [20, 67, 68]. In other words, the more diverse communities contained multiple populations with redundant phenotypes that allow for normal community-aggregated function to persist even if some of those populations were lost following stress. It is also possible that α diversity played other indirect roles in supporting ecosystem functions and stability, e.g., by altering ecological interactions within and among taxa. These indirect influences, along with the observation that only low α diversity soils exhibited reduced stability, suggest that bacterial α diversity may be a useful and easily generalizable indicator of the functioning and stability of soil ecosystems. However, our results also suggest that bacterial community membership (i.e., ASV composition) better reflects the direct microbial drivers of ecosystem function, though this metric is difficult to generalize across environmental contexts. Our results also show, however, that coarser taxonomic information can be informative – for example, phylum-level taxonomy was a strong predictor of biomass-specific respiration (qCO_2_) and genus-level taxonomy improved some models (SIR and qCO_2_) and suggested important C-cycle functions for *Pseudomonas* and *Bacillus*. Our results also demonstrated that information about specific functional groups will be important for predicting ecosystem functions that are performed by phylogenetically narrow groups [68], evidenced by the strong relationship observed between nitrification rates and nitrifier abundance. Overall, our study demonstrates the central importance of community membership for supporting ecosystem functioning and stability and identifies some specific bacterial taxa that are important for predicting ecosystem responses. Nevertheless, identifying generalizable aspects of microbial community membership that are informative at the ecosystem scale remains a challenge in microbial ecology [2].

While our focus was on influences of bacterial diversity on ecosystem stress responses, we also observed that stress responses were distinct between the two ecosystem types. Specifically, C-cycle metrics (e.g., SIR, qCO_2_) were more impacted by stress in prairie soils while N-cycle metrics (i.e., N mineralization, nitrification) were more impacted by stress in cultivated soils. This further demonstrates that ecosystem responses to anthropogenic stress are strongly dependent upon microbial community membership – these two ecosystems hosted different initial bacterial communities and thus exhibited distinct responses to stress. The stress responses of the prairie soils were also larger in magnitude, particularly when examining overall ecosystem functioning, i.e., multifunctionality. This contrasts with our original hypothesis that the more diverse communities present in the prairie soils would confer greater ecosystem stability and suggests that historical exposure to stress (i.e., our cultivated soils) may select for a more stress-tolerant community that is more resistant to future stressors. An alternative explanation is that the historical stress applied to the cultivated soils has resulted in low biomass communities (Supplementary Fig. S10) that already exhibit minimal function with respect to many ecosystem processes and therefore no further stress response is possible. Regardless, our results suggest that high-functioning natural ecosystems may also be the most vulnerable to anthropogenic stress.

Many of the stress responses we observed were expected given that the stressor we applied, oxytetracycline, is a bacteriostatic antibiotic that inhibits bacterial activity by preventing protein production. For example, stress reduced SIR and N mineralization rates, two processes that are dependent upon overall microbial activity. We also observed increased qCO_2_ (lower efficiency) following antibiotic application, which has also been observed in prior studies [69, 70]. This may reflect increased maintenance demands of bacteria and/or shifts towards less efficient bacterial taxa. Antibiotic stress also significantly increased hydrolytic enzyme activity in D2 soils from the prairie ecosystem, which indicates greater allocation of resources to C-, N-, and P-acquisition in these communities. Greater allocation of cellular resources towards enzyme production as opposed to biomass production is in accordance with the reduced efficiency (higher qCO_2_) we observed in these soils [71]. Interestingly, however, the abundance of tetracycline antibiotic resistance genes (ARGs) was not a strong predictor of ecosystem processes, despite the fact that ARG abundance was indeed low or undetectable in the D2 soils that also exhibited the greatest stress responses (Supplementary Fig. S16). It is possible that other tet ARGs (e.g., tetO, tetX) that we did not measure played important roles in our experiment. Alternatively, it is likely that different microbial taxa exhibit different degrees of intrinsic resistance to this antibiotic or distinct recovery patterns following application of this stress, thus accounting for the patterns we observed.

It is also important to note that our study did not consider influences of eukaryotic organisms (e.g., fungi, protists) as our experimental approach proved unsuitable for manipulation of those communities. Despite this limitation, the lack of fungal establishment in our microcosms has the benefit of allowing us to more completely isolate the influences of bacteria (the direct targets of most antibiotics) on ecosystem function and stability. However, multiple prior studies have provided evidence of eukaryotic influences on ecosystem functions, including significant statistical associations between eukaryotic α diversity and soil processes [15, 16]. Therefore, we recommend that future studies attempt to comprehensively assess characteristics of eukaryotic communities that support soil ecosystem function and stability, similar to what we have done here for bacterial communities. Future studies should also incorporate additional dimensions of soil function (e.g., plant productivity, pathogen suppression) when quantifying multifunctionality, as these functions may exhibit different relationships with microbial communities compared with the biogeochemical functions measured here.

Regardless, the central conclusions of our study are clear – that microbial abundance and bacterial community membership, and not α diversity, emerge as the strongest predictors of soil ecosystem processes. This conclusion is supported by re-analysis of a prior microbial diversity – ecosystem stress experiment [23], which yielded similar results to ours, thus reinforcing our findings. Indeed, the observation of similar patterns in soils from distinct biogeographic regions and across a broad range of global change factors demonstrates that our results are robust across ecological contexts. Overall, our results demonstrate that while bacterial α diversity may be a simple and useful indicator of ecosystem function and stability, microbial abundance and community membership metrics are stronger statistical predictors of function and more likely reflect the biological mechanisms by which microbial communities influence ecosystems. We suggest that future studies also comprehensively examine all possible microbial drivers of ecosystem function as opposed to considering influences of α diversity alone. Continued development of this line of research is critical – microorganisms are the proximate drivers of nearly all ecosystem functions [2] and will determine the fate of ecosystems in the face of intensifying anthropogenic stress. Therefore, it is imperative to identify microbial characteristics that will influence ecosystem stress responses. We demonstrate that variation in microbial abundance and community membership are the dominant drivers of ecosystem processes and are therefore of primary importance when seeking to understand and predict ecosystem responses to global change.

## Supplementary information

Supplementary Methods

## Data Availability

Raw sequence data is deposited in the NCBI archive under accession number PRJNA853373. All other data and reproducible R code are available on figshare: 10.6084/m9.figshare.21513366.v3
