# Supplementary material for: Evaluating the role of bacterial diversity in supporting soil ecosystem functions under anthropogenic stress
Source: ISME Commun. 2023 Jul 3;3:66. doi: 10.1038/s43705-023-00273-1 (PMC10318037; doi:10.1038/s43705-023-00273-1)
Supplement: Supplementary file 1 — Supplementary Methods [file 43705_2023_273_MOESM1_ESM.docx]

**Evaluating the role of bacterial diversity in supporting soil ecosystem functions under anthropogenic stress**

**Supplementary Methods**

***Soil chemical and physical analyses***

We determined gravimetric water content by mass loss after drying field-moist samples at 105 ℃ for 24 h and water holding capacity by wetting soils to field capacity and then drying at 105 ℃ for 24 h. On the two original nonsterile soils, we determined pH in a 1:1 soil:DI H_2_O slurry using a SevenCompact pH meter (Metter Toledo, Columbus OH,USA) and total C and N using a an ECS 4010 elemental analyzer (Costech Inc., Santa Clarita, CA, USA). On all soils (original and experimental), we performed an extraction with 0.5 M K_2_SO_4_ (1:5 soil:solution ratio) and analyzed extracts for extractable organic C (DOC) and total extractable N (TDN) on a Shimadzu TOC-L TNM-L analyzer (Shimadzu Instruments, Kyoto, Japan). Extracts were also analyzed for NH_4_-N using the phenol-hypochlorite method [1] and NO_3_-N using the vanadium reduction method [2].

***qPCR and Amplicon Sequencing***

To measure total 16S rRNA and ITS gene abundances, we performed qPCR using the Eub 338/518 primer pair and ITS1f/5.8s primer pairs, respectively [3]. Thermal cycling conditions for 16S and ITS were 15 min at 95 °C followed by 40 cycles of 15 s at 94 °C, 30 s at 55 °C and 30 s at 72 °C. To measure abundance of ammonia-oxidizing bacteria (AOB), we used the primer pair amoA-1f/2r[4] and thermal cycling protocols were 2 min at 95 °C followed by 40 cycles of 15 s at 94 °C, 30 s at 55 °C, 60 s at 72 °C, and 5s at 78 °C (plate read step). To measure abundance of ammonia-oxidizing archaea (AOA), we used the primer pair Arch-amoA f/r[5] and thermal cycling protocols were 2 min at 95 °C followed by 40 cycles of 15 s at 94 °C, 30 s at 53 °C, and 60 s at 72 °C. We used previously described primer sets to amplify two tetracycline resistance genes: tetM[6] and and tetW[7]. For both tet genes, thermal protocols were 2 min at 95 °C followed by 40 cycles of 15 s at 95 °C, 30 s at 53 °C, and 60 s at 72 °C. Because the tet genes were often individually undetectable (especially in reduced diversity soils), we summed the abundances of the two tet genes in each sample for all downstream analyses. All qPCR reactions contained 10 μl PowerUp SYBR green master mix (Applied Biosystems, Waltham, MA, USA), 0.5 μM (16S, ITS, tetM, tetW) or 0.25 μM (AOA, AOB) forward and reverse primer, 2 μl DNA template, and nuclease-free H_2_O to 20μl. Standard curves were generated by amplifying serial dilutions of plasmids containing cloned copies of the target sequences. All qPCR reactions were performed in triplicate and amplification specificity was verified using melt curve analysis. All qPCR runs had R^2^ values > 0.99 and efficiency > 80%.

Bacterial community diversity and composition was determined by amplicon sequencing of V4 region of the 16S rRNA gene using the primer pair 515F/806R[8]. PCR reactions contained 10 μl Thermo Fisher Platinum II Hot Start PCR Master Mix (Thermo Fisher Inc., Waltham, MA, United States), 1 μl DNA template, 0.2 μM forward and reverse primer, and nuclease free H_2_O to 25 μl. We also amplified blanks to detect possible contamination. Thermal cycling conditions were 2 min at 94℃ followed by 35 cycles of 45 s at 94℃, 60 s at 50℃, and 90 s at 72℃, with a 10 min final extension at 72℃. We visualized amplicons and negative controls on an agarose gel, quantified amplicons using a Qubit Fluorometer (Thermo Fisher Inc., Waltham, MA, United States), and pooled amplicons in equimolar ratios. Amplicons were sequenced on an Illumina MiSeq using 250bp paired-end reads. Raw reads were deposited in the NCBI archive under accession number PRJNA853373.

We processed raw sequences using DADA2[9] and assigned taxonomy to unique sequences (i.e., ASVs) using the RDP classifier[10] trained on the SILVA database (version 138.1)[11]. After processing, we rarefied samples to 15,418 sequences per sample to account for differences in sequence depth among samples. To assess changes in bacterial life history across treatments, we used rrndb[12] to estimate the rRNA operon number for each unique sequence and then calculated the average rRNA operon number per organism for each sample by calculating the product of operon number and ASV relative abundance, summed for all ASVs in a sample[13, 14].

**Supplementary Tables and Figures**

**Table S1:** Pre-treatment soil variables from cultivated and prairie sites. Values are means of three independently prepared subsamples of the original composite sample from each site.

| **Variable** | **Cultivated** | **Prairie** |
| --- | --- | --- |
| Moisture (g H_2_O gdw^-1^) | 0.241 | 0.288 |
| pH | 5.58 | 6.84 |
| NO_3_^-^ (µg N gdw^-1^) | 4.43 | 2.38 |
| NH_4_^+^ (µg N gdw^-1^) | 1.66 | 3.07 |
| Total Extractable N (µg N gdw^-1^) | 10.90 | 9.42 |
| Extractable DOC (µg C gdw^-1^) | 95.1 | 89.4 |
| Extractable C:N | 8.73 | 9.48 |
| Total C (mg C gdw^-1^) | 15.5 | 23.2 |
| Total N (mg N gdw^-1^) | 1.35 | 1.81 |
| Total C:N | 11.5 | 12.8 |


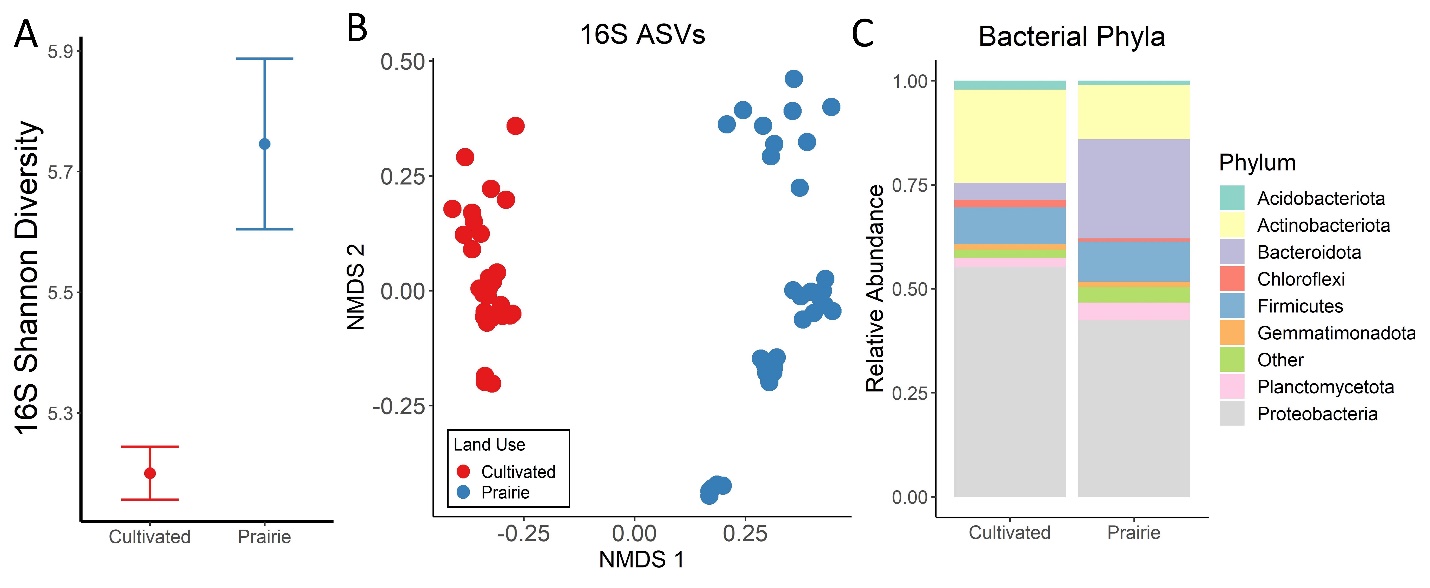


Figure S1: Cultivated and prairie sites host distinct resident microbial communities in terms of bacterial α diversity (A), 16S sequence-level community composition (B) and bacterial phylum-level composition (C).


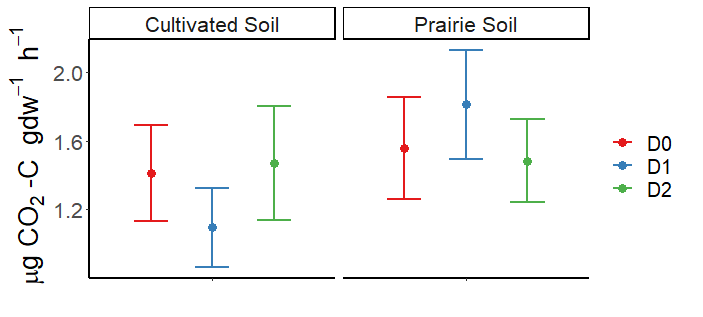


Figure S2: Respiration data at the end of the six-week community establishment period but prior to the application of antibiotic stress. These data show that dilution treatments within each land use exhibited indistinguishable respiration (Dilution treatment *P* > 0.05), indicating that microbial activity had recovered prior to stress application.


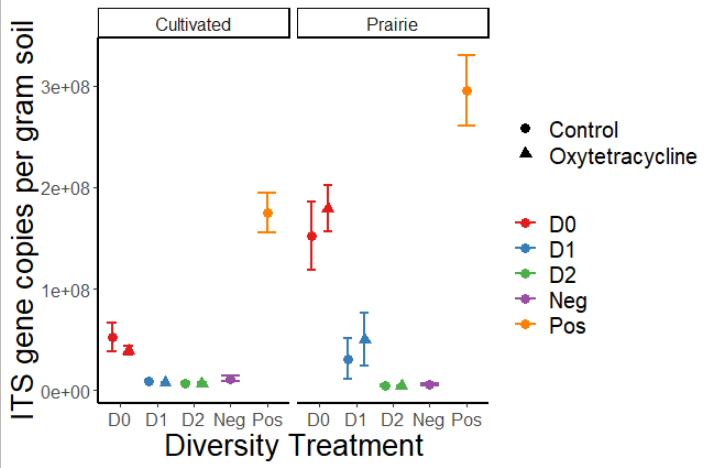


Figure S3: ITS gene copy abundance across dilution treatments. These results indicate extremely low fungal abundance in the dilution treatments, particularly D1 and D2. This indicates poor fungal establishment in the microcosms, likely due to few, if any, fungal cells present in the D1 and D2 inocula (based on ITS abundance per gram of soil in the original soils and the soil mass-equivalent of the D1 and D2 inocula).


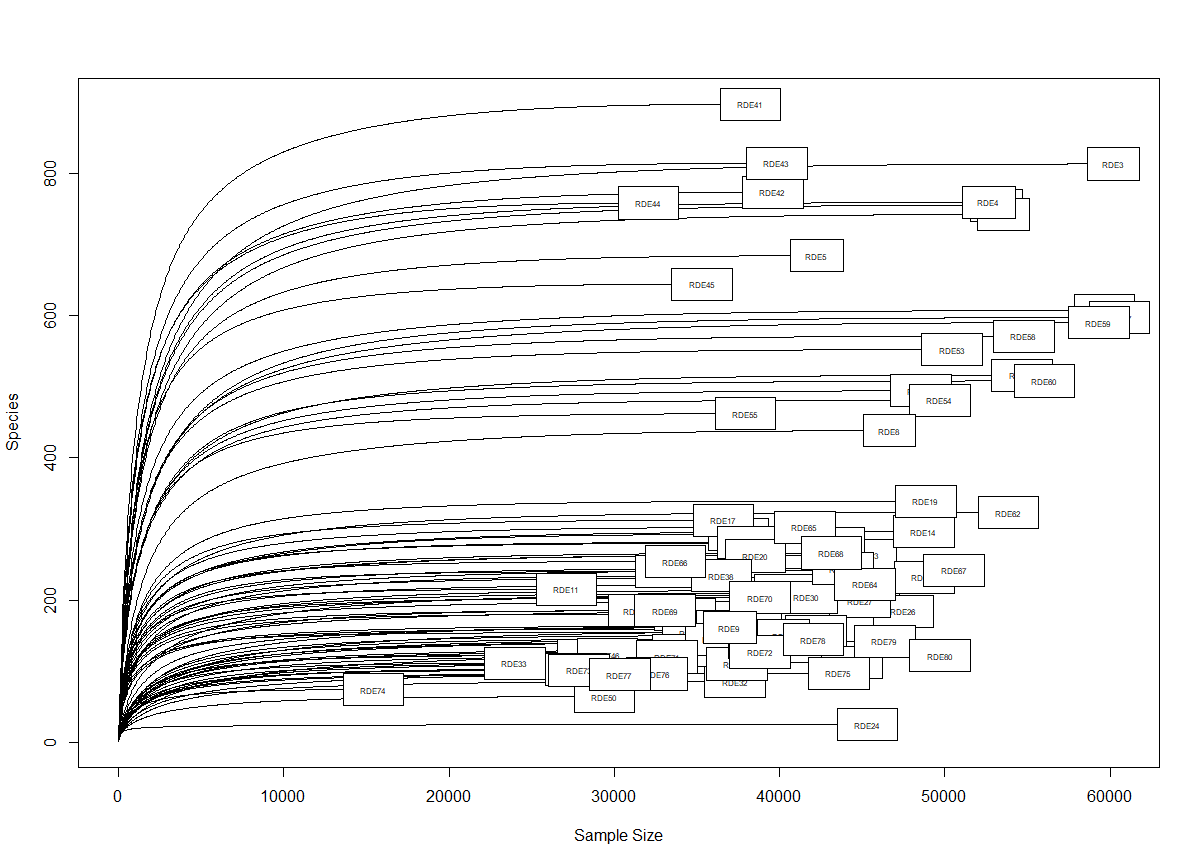


Figure S4: Rarefaction curves for all samples indicate adequate coverage at 15,418 sequences per sample.


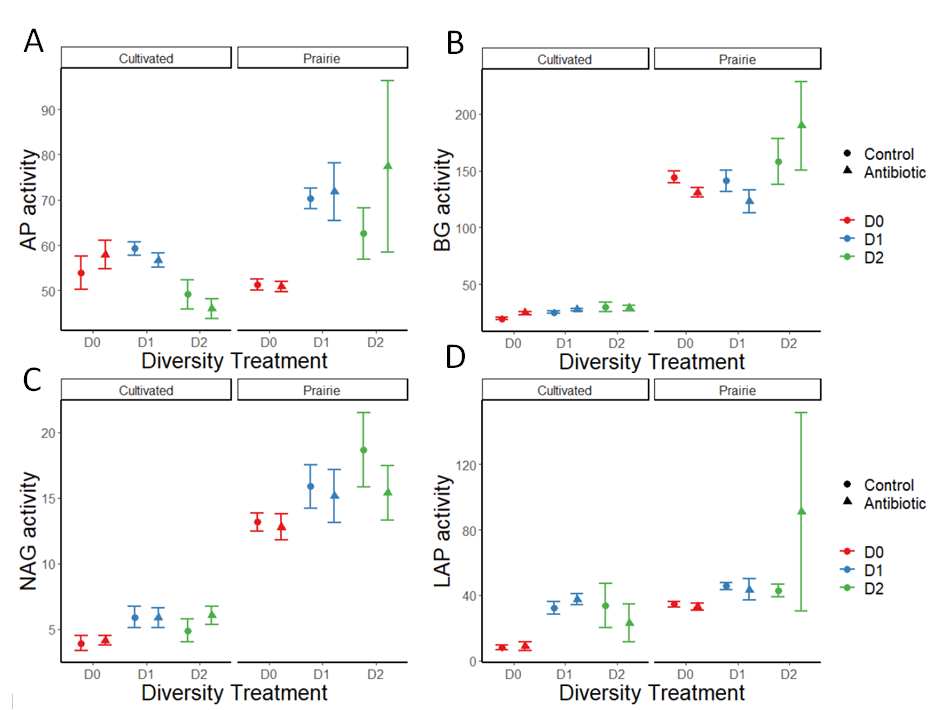


Figure S5: Individual enzyme activities across treatments, including AP (A), BG (B), NAG (C), and LAP (D). Units are in nmol per gram of dry soil per hour. Activities are generally higher in prairie communities and were generally increased by antibiotic additions in the low diversity (D2) prairie community.


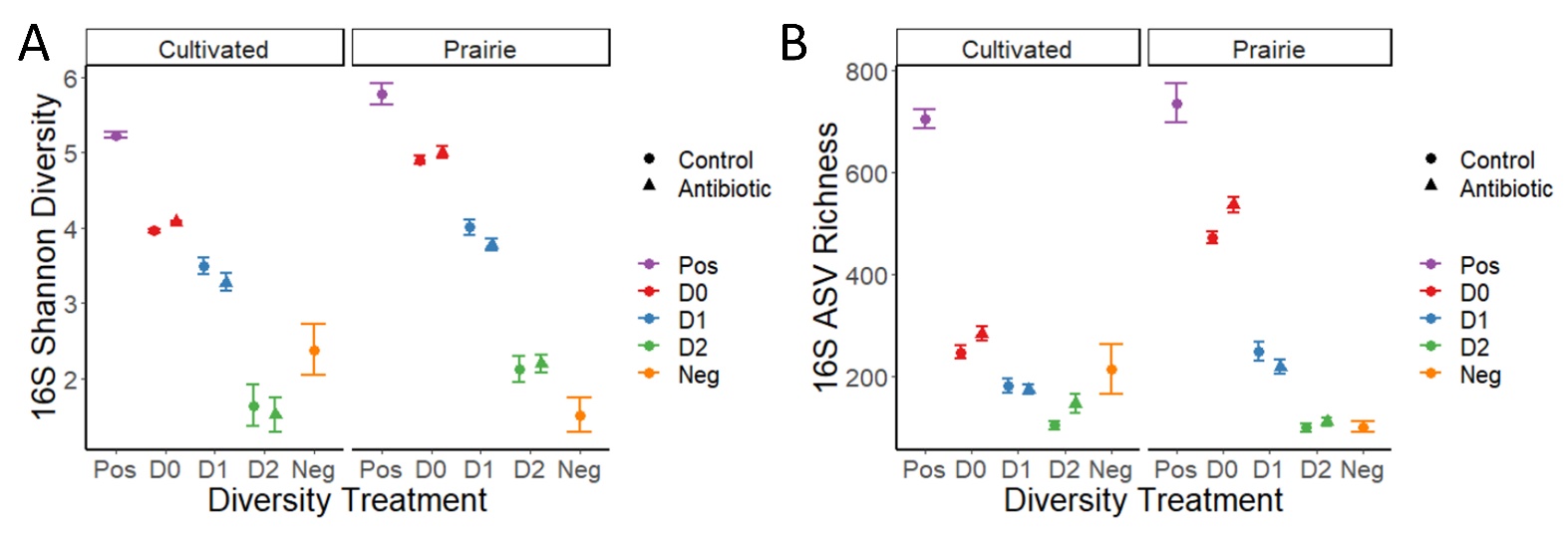


Figure S6: 16S Shannon diversity (A) and ASV Richness (B) across all experimental groups, including the original nonsterile soils (Pos) and sterile soils that were inoculated only with water (Neg).


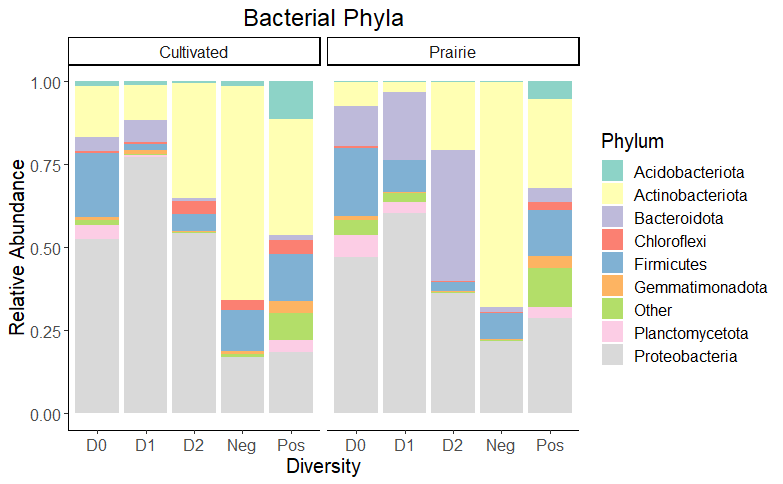


Figure S7: Bacterial community composition among diversity treatments. Increasing dilutions (i.e., decreasing diversity) resulted in increasing dissimilarity from the positive controls but remained distinct from the negative controls.


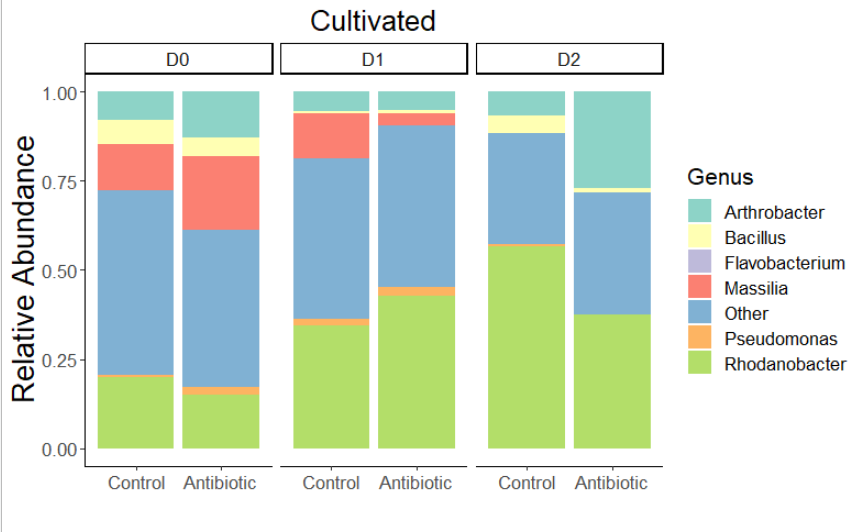


Supplementary Figure S8: Relative abundances of bacterial genera identified by DESeq2 as differentially abundant among treatments (cultivated soils). Effects of stress on *Bacillus* and *Arthrobacter* only occurred in D2 soils, while *Massilia* responded to stress in D1 soils.


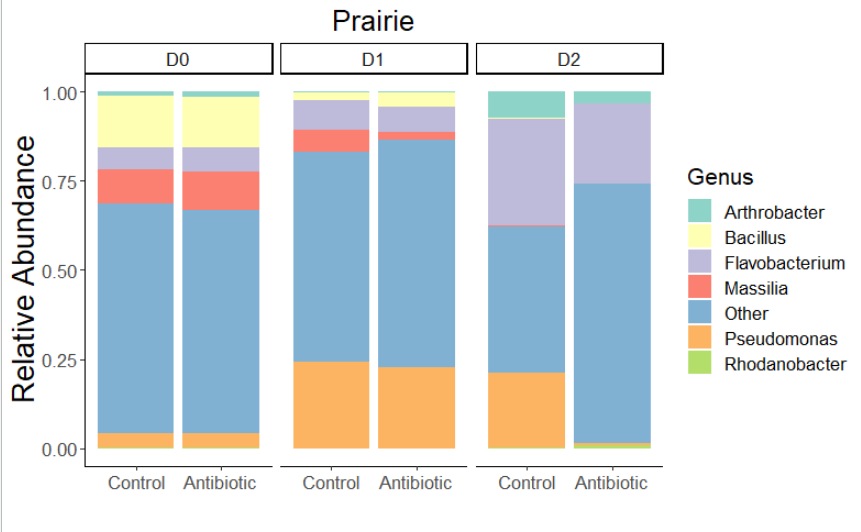


Supplementary Figure S9: Relative abundances of bacterial genera identified by DESeq2 as differentially abundant among treatments (prairie soils). Effects of stress on *Pseudomonas* only occurred in D2 soils, while *Massilia* responded to stress in D1 soils.


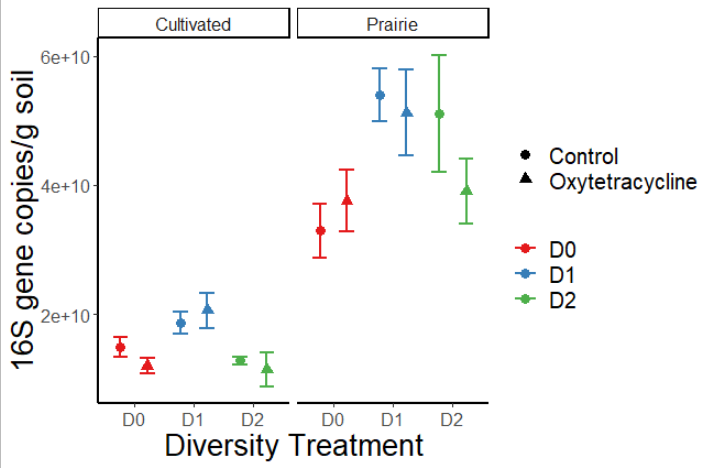

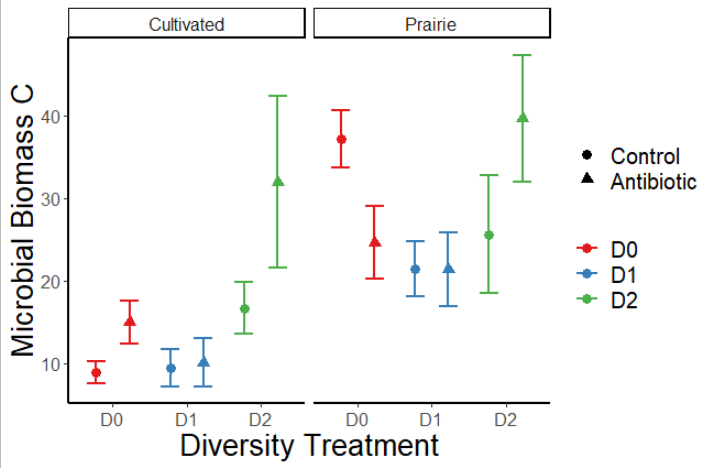

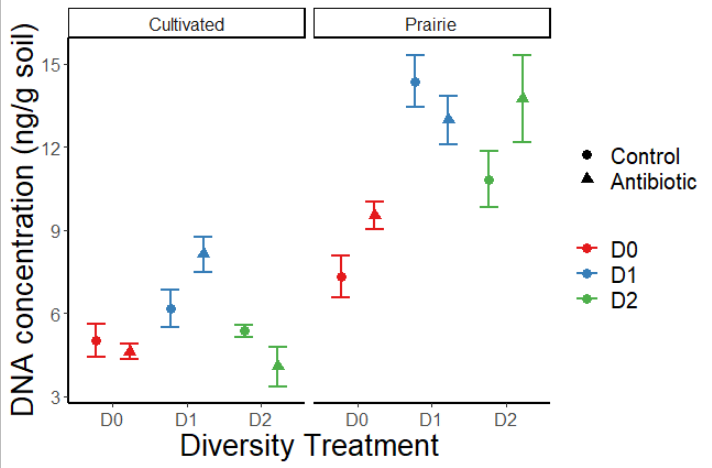


Figure S10: Microbial abundance metrics across treatments. Units for microbial biomass C are in µg per gram of soil. 16S gene copies were not affected by stress treatments. Biomass metrics generally increased following stress in D2 soils – MBC was significantly higher in stressed D2 soils from the cultivated ecosystem while DNA was significantly higher in stressed D2 soils from the prairie.


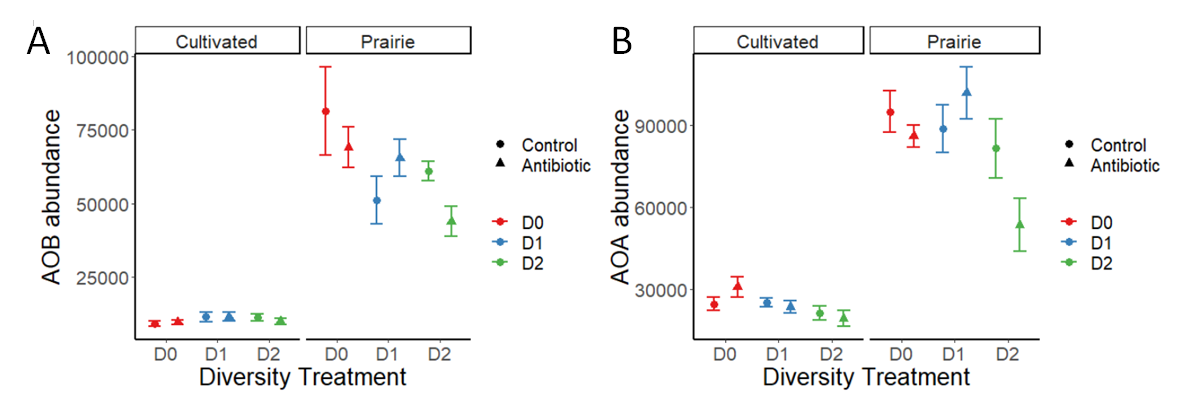


Figure S11: Abundance of ammonia-oxidizing bacteria (AOB) and ammonia-oxidizing archaea (AOA) across treatments. Units are gene copies per gram of dry soil.


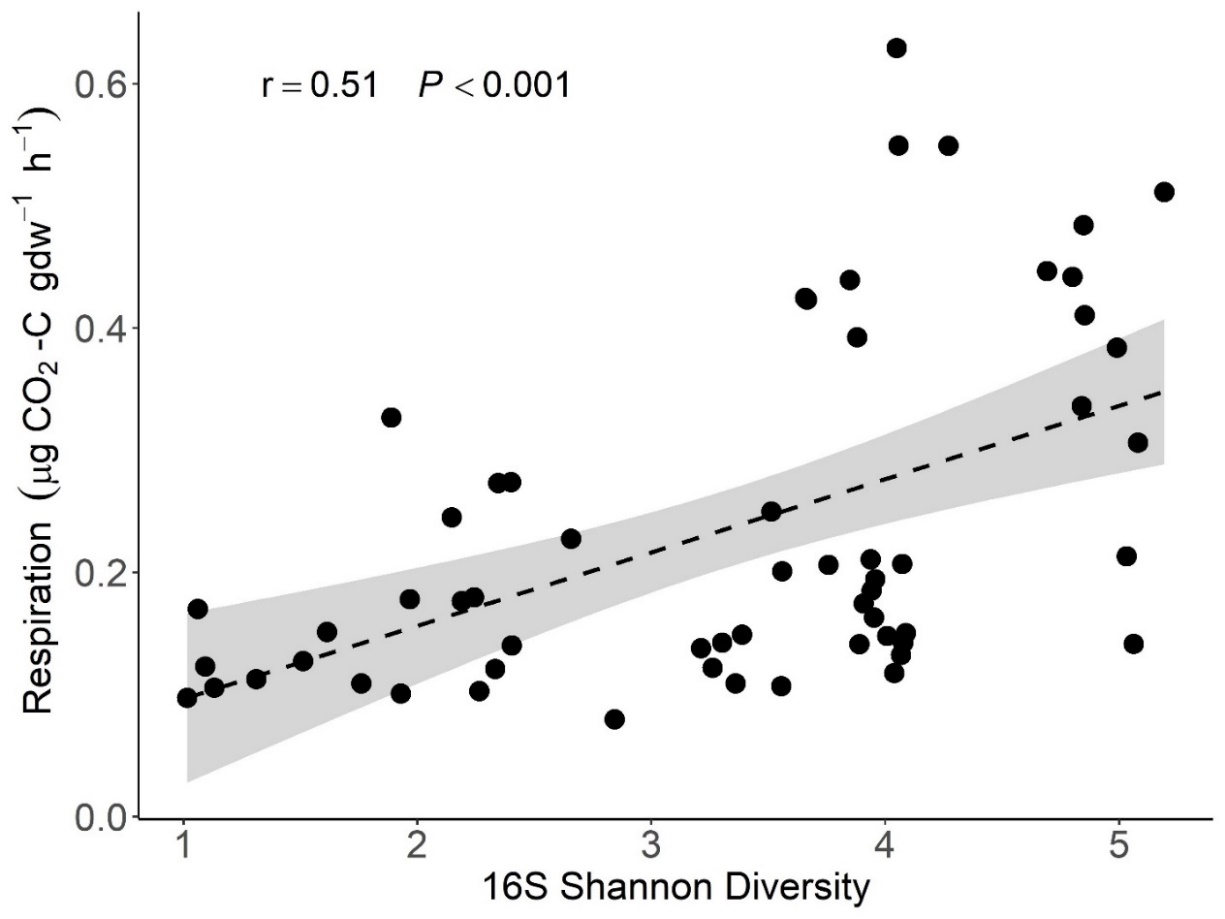


Figure S12: Relationship between bacterial Shannon diversity and respiration (C mineralization) rates.


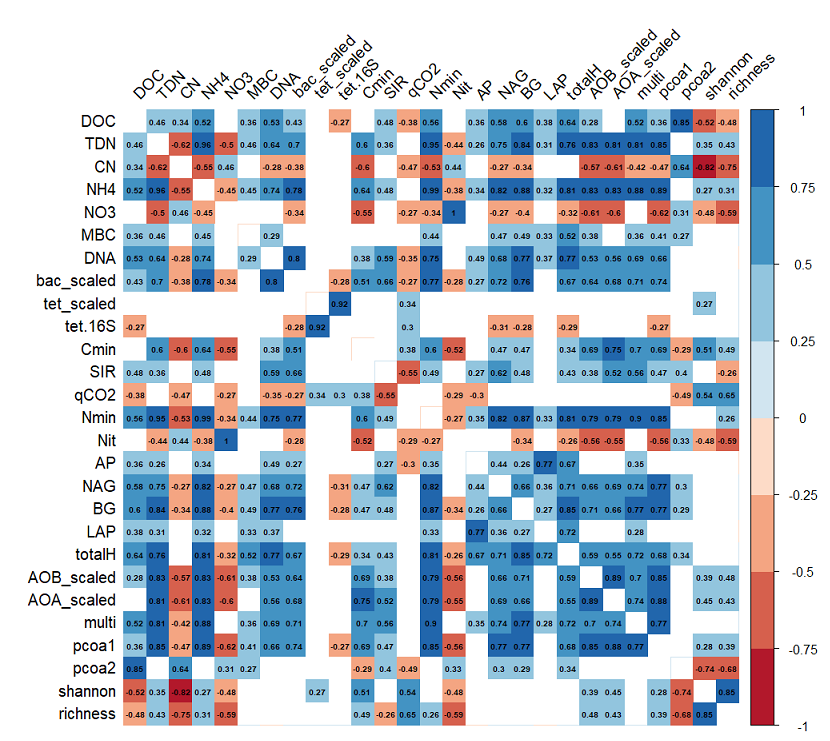


Figure S13: Pearson correlations among all variables measured in this study. Abundance metrics: ‘MBC’ is microbial biomass carbon (µg per gram of soil), ‘DNA’ is DNA yield (ng per gram of soil) and ‘bac_scaled’ is 16S gene copies per gram of soil. Statistically significant correlations (*P* < 0.05) are colored in. Note the lack of correlations between abundance metrics and alpha diversity metrics (‘shannon’ and ‘richness’).


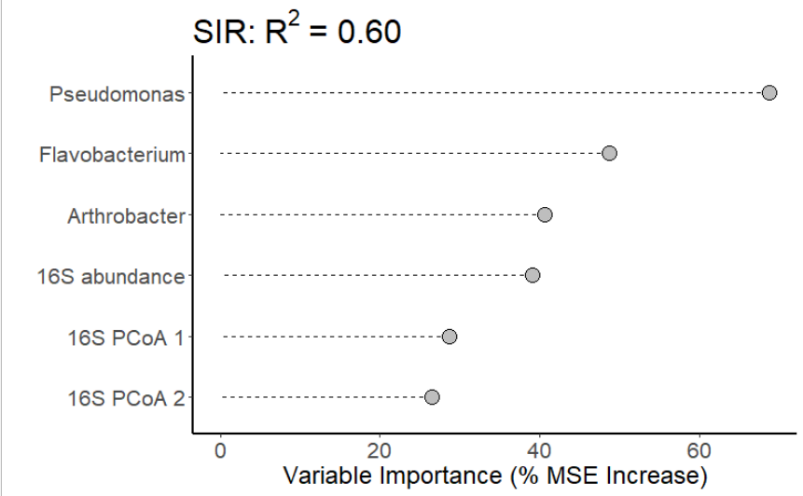

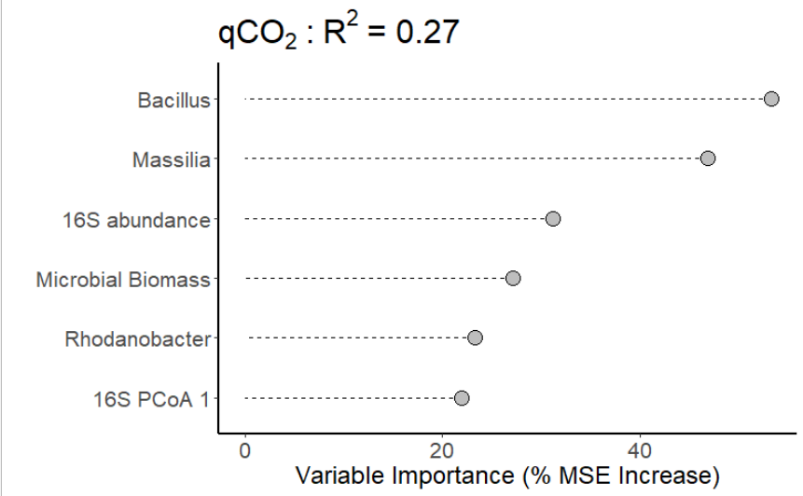


Figure S14: Random forest models for SIR and qCO2 using differentially abundant genera as predictors. Models for all other processes were not improved by including genus-level taxonomy.


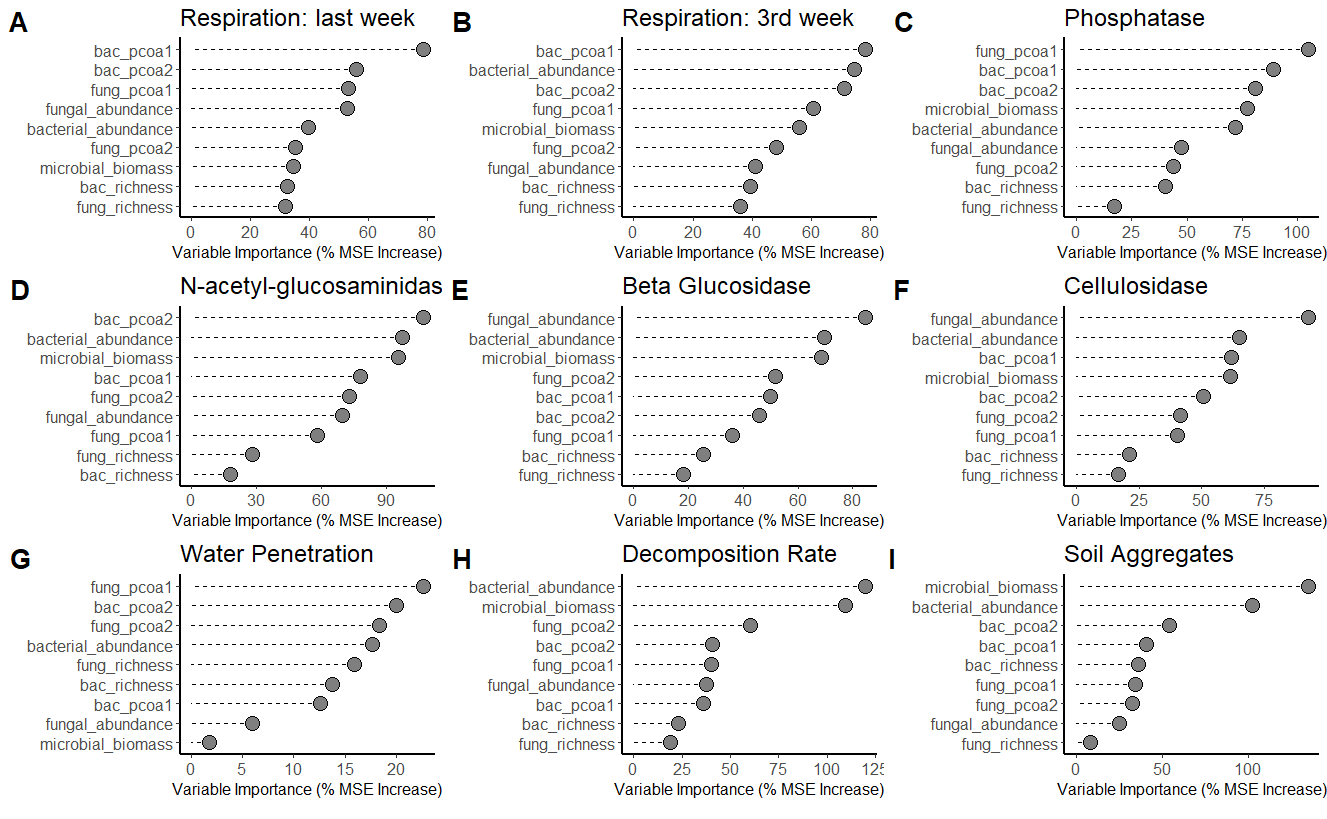


Figure S15: Random forest analysis of data from Yang et al. 2022[17]. The ‘Importance’ of microbial predictor variables for each ecosystem function was calculated by determining the increase in model error after randomly shuffling each candidate predictor across the data set.


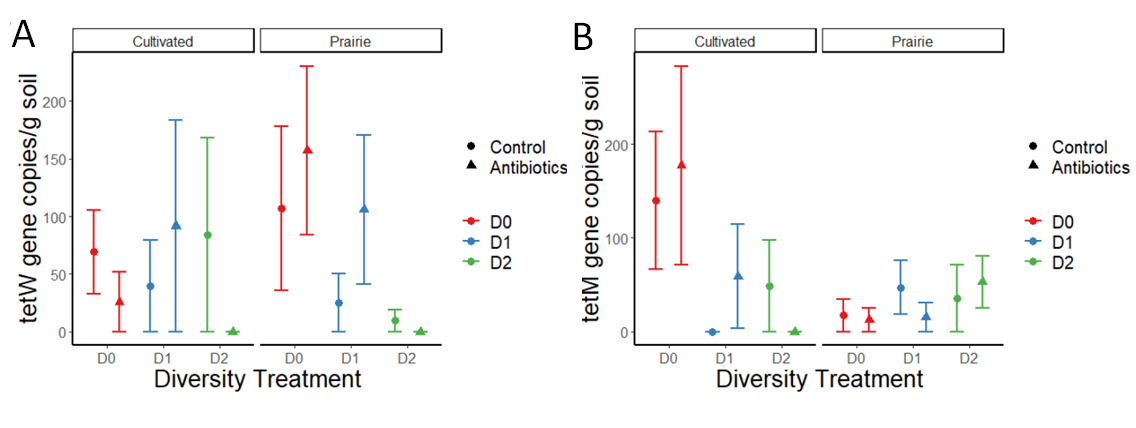


Figure S16: Abundance of tetM (A) tetW (B) tetracycline antibiotic resistance genes across treatments. Units are gene copies per gram of dry soil.

**References**

1. Weatherburn M. Phenol-hypochlorite reaction for determination of ammonia. *Anal Chem* 1967; **39**: 971–974.

2. Doane TA, Horwáth WR. Spectrophotometric Determination of Nitrate with a Single Reagent. *Analytical Letters* 2003; **36**: 2713–2722.

3. Fierer N, Jackson JA, Vilgalys R, Jackson RB. Assessment of Soil Microbial Community Structure by Use of Taxon-Specific Quantitative PCR Assays. *Appl Environ Microbiol* 2005; **71**: 4117–4120.

4. Rotthauwe JH, Witzel KP, Liesack W. The ammonia monooxygenase structural gene amoA as a functional marker: molecular fine-scale analysis of natural ammonia-oxidizing populations. *Appl Environ Microbiol* 1997; **63**: 4704–4712.

5. Francis CA, Roberts KJ, Beman JM, Santoro AE, Oakley BB. Ubiquity and diversity of ammonia-oxidizing archaea in water columns and sediments of the ocean. *Proc Natl Acad Sci USA* 2005; **102**: 14683–14688.

6. Ng L-K, Martin I, Alfa M, Mulvey M. Multiplex PCR for the detection of tetracycline resistant genes. *Molecular and Cellular Probes* 2001; **15**: 209–215.

7. Aminov RI, Garrigues-Jeanjean N, Mackie RI. Molecular Ecology of Tetracycline Resistance: Development and Validation of Primers for Detection of Tetracycline Resistance Genes Encoding Ribosomal Protection Proteins. *Applied and Environmental Microbiology* 2001.

8. Walters W, Hyde ER, Berg-Lyons D, Ackermann G, Humphrey G, Parada A, et al. Improved Bacterial 16S rRNA Gene (V4 and V4-5) and Fungal Internal Transcribed Spacer Marker Gene Primers for Microbial Community Surveys. *mSystems* 2016; **1**: e00009-15.

9. Callahan BJ, McMurdie PJ, Rosen MJ, Han AW, Johnson AJA, Holmes SP. DADA2: High-resolution sample inference from Illumina amplicon data. *Nat Methods* 2016; **13**: 581–583.

10. Wang Q, Garrity GM, Tiedje JM, Cole JR. Naïve Bayesian Classifier for Rapid Assignment of rRNA Sequences into the New Bacterial Taxonomy. *Applied and Environmental Microbiology* 2007; **73**: 5261–5267.

11. Quast C, Pruesse E, Yilmaz P, Gerken J, Schweer T, Yarza P, et al. The SILVA ribosomal RNA gene database project: improved data processing and web-based tools. *Nucleic Acids Res* 2013; **41**: D590–D596.

12. Stoddard SF, Smith BJ, Hein R, Roller BRK, Schmidt TM. rrnDB: improved tools for interpreting rRNA gene abundance in bacteria and archaea and a new foundation for future development. *Nucleic Acids Research* 2015; **43**: D593–D598.

13. Veach AM, Zeglin LH. Historical Drought Affects Microbial Population Dynamics and Activity During Soil Drying and Re-Wet. *Microb Ecol* 2020; **79**: 662–674.

14. Osburn ED, McBride SG, Kupper JV, Nelson JA, McNear DH, McCulley RL, et al. Accurate detection of soil microbial community responses to environmental change requires the use of multiple methods. *Soil Biology and Biochemistry* 2022; **169**: 108685.

15. Zhou Z, Wang C, Luo Y. Effects of forest degradation on microbial communities and soil carbon cycling: A global meta-analysis. *Global Ecol Biogeogr* 2018; **27**: 110–124.

16. Osburn ED, McBride SG, Aylward FO, Badgley BD, Strahm BD, Knoepp JD, et al. Soil Bacterial and Fungal Communities Exhibit Distinct Long-Term Responses to Disturbance in Temperate Forests. *Front Microbiol* 2019; **10**.

17. Yang G, Ryo M, Roy J, Lammel DR, Ballhausen M-B, Jing X, et al. Multiple anthropogenic pressures eliminate the effects of soil microbial diversity on ecosystem functions in experimental microcosms. *Nat Commun* 2022; **13**: 4260.
